# Salivary Antibodies against Multiple Environmental Pathogens Found in Individuals Recreating at an Iowa Beach

**DOI:** 10.3390/ijerph18115797

**Published:** 2021-05-28

**Authors:** Swinburne A. J. Augustine, Tarsha N. Eason, Tim Wade, Shannon M. Griffin, Elizabeth Sams, Kaneatra Simmons, Malini Ramudit, Kevin Oshima, Alfred Dufour

**Affiliations:** 1 Center for Public Health and Environmental Assessment, United States Environmental Protection Agency, Cincinnati, OH 45268, USA; griffin.shannon@epa.gov; 2 Center for Environmental Methods and Measurement, United States Environmental Protection Agency, Athens, GA 30605, USA; eason.tarsha@epa.gov; 3 Center for Public Health and Environmental Assessment, United States Environmental Protection Agency, Research Triangle Park, NC 27709, USA; wade.tim@epa.gov (T.W.); sams.elizabeth@epa.gov (E.S.); 4 Department of Arts and Sciences/Learning Support, Fort Valley State University, Fort Valley, GA 31030, USA; kaneatra.simmons@fvsu.edu; 5 Oak Ridge Institute for Science Education, Oak Ridge, TN 37831, USA; ramudimk@gmail.com; 6 Center for Environmental Methods and Measurement, United States Environmental Protection Agency, Cincinnati, OH 45268, USA; oshima.kevin@epa.gov (K.O.); dufour.alfred@epa.gov (A.D.)

**Keywords:** saliva, multiplex, immunoassay, immunoprevalence, immunoconversion, incident infection, coinfection, waterborne, environmental pathogens, Luminex, population surveillance, public health, recreational beach, Iowa, Buffalo Shores Beach

## Abstract

Detecting environmental exposures and mitigating their impacts are growing global public health challenges. Antibody tests show great promise and have emerged as fundamental tools for large-scale exposure studies. Here, we apply, demonstrate and validate the utility of a salivary antibody multiplex immunoassay in measuring antibody prevalence and immunoconversions to six pathogens commonly found in the environment. The study aimed to assess waterborne infections in consenting beachgoers recreating at an Iowa riverine beach by measuring immunoglobulin G (IgG) antibodies against select pathogens in serially collected saliva samples. Results showed that nearly 80% of beachgoers had prior exposures to at least one of the targeted pathogens at the beginning of the study. Most of these exposures were to norovirus GI.1 (59.41%), norovirus GII.4 (58.79%) and *Toxoplasma gondii* (22.80%) and over half (56.28%) of beachgoers had evidence of previous exposure to multiple pathogens. Of individuals who returned samples for each collection period, 6.11% immunoconverted to one or more pathogens, largely to noroviruses (GI.1: 3.82% and GII.4: 2.29%) and *T. gondii* (1.53%). Outcomes of this effort illustrate that the multiplex immunoassay presented here serves as an effective tool for evaluating health risks by providing valuable information on the occurrence of known and emerging pathogens in population surveillance studies.

## 1. Introduction

Waterborne, foodborne and environmentally transmitted infections continue to be a serious global concern for both developed and developing countries [1]. A prominent area of concern is the well-documented association between fecal contamination and the risk of gastrointestinal (GI) illness for individuals recreating in oceans and lakes. However, much less is known about the health risks associated with swimming in inland rivers [2]. Large inland rivers are a valuable source for recreation, but they receive discharges from numerous sources including treated and untreated sewage, wastewater and contaminated runoff that may cause acute health effects (e.g., infections). Since some infections present without observable symptoms, immunological responses can be used to identify the etiological agents and estimate both symptomatic and asymptomatic disease burden [3]. The detection of asymptomatic infections, especially asymptomatic chronic viral infections, is vital for elucidating the transmission of pathogenic infections and estimating the burden of disease [4,5]. Immunoassays examine circulating antibodies against specific pathogens as biological markers of infection. Assays using noninvasive samples (e.g., saliva) are particularly appealing because their ease of collection, storage and use make it easier to recruit study participants, especially children. Recent studies suggest that saliva may in some cases be an appropriate alternative biofluid to serum for detecting antibodies [6,7,8,9,10,11,12].

In previous work, we described the development and utility of salivary antibody multiplex immunoassays in measuring symptomatic and asymptomatic infections, immunoprevalence, coinfections and incident infections associated with recreating in contaminated waters and other environmental and water-related exposures [13,14,15,16,17,18]. These research efforts demonstrated that the immunoassays could provide cost and time savings in comparison to traditional enzyme-linked immunosorbent assays (ELISAs) as more analytes are added to the assay [18,19].

While previous studies concentrated on marine beachgoers, this study focuses on investigating the prevalence of exposure and incident infections in a riverine beachgoing community by examining the presence of IgG antibodies against six pathogens: norovirus genotypes GI.1 and GII.4, *Helicobacter pylori*, hepatitis A virus [HAV], *Toxoplasma gondii* and *Campylobacter jejuni*). These six pathogens were selected because of the public health implications associated with infections and although they may have multiple routes of transmission, all have been identified as possible sources of waterborne disease. Moreover, immunogenic antigens from these pathogens were readily available commercially. A more detailed description for the choice of these pathogens can be found in Augustine et al., 2017 [13] (p. 2). The study was conducted at Buffalo Shores Beach, a large recreational area in the city of Buffalo, Iowa because it is located downstream of multiple wastewater treatment plants that are believed to contribute to water contamination and waterborne infections in individuals recreating there, and the beach is well-populated in the summer months.

## 2. Materials and Methods

This study followed a similar sampling protocol and analytical design to previously conducted studies. In brief, this study used a multiplex Luminex platform to detect salivary antibody responses to antigens (Ag) as shown in Table 1 [6,13,15,16]. Table 1 provides information on the organism, antigen, source of where the antigen was purchased/acquired, and the amount of antigen coupled to the beads. *T. gondii* Recombinant p30 (SAG1) in Table 1 refers to Surface Antigen 1.

### 2.1. Collection, Processing and Analysis of Saliva Samples

In the summer of 2011, we collected saliva samples from 481 consenting individuals who recreated at Buffalo Shores Beach, Iowa. Study participants ranged in age from 1 to 73 years, with a mean of 22 years and 57% were female. Most respondents were White (77%) and 26% reported Hispanic ethnicity. All individuals provided informed consent before enrolling and participating in the study. The Institutional Review Board of the University of North Carolina, Chapel Hill, NC, USA (IRB # 11-0737) reviewed and approved the protocol and procedures. Saliva samples were obtained by rubbing an Oracol™ sponge sampler (Malvern Medical Developments, Worcester, UK) against the crevices in the gingival space between the gums and teeth. Due to the potential for contamination by maternal antibodies and high rates of non-waterborne infections, infants younger than one year old were excluded from the study. Further, to participate in the study, individuals had to be able to read and write English or Spanish, and at least one household member had to be over 18 years of age.

Three crevicular saliva samples were collected from study subjects as follows: trained study staff collected the baseline (S1) samples at the beach between the hours of 9:00 a.m. and 5:00 p.m. S2 and S3 samples were collected at home by the participants as instructed 10 days and 40 days, respectively, post baseline sample collection. The self-collected samples were shipped next day service on ice to the laboratory for processing and storage. On receipt of the samples, they were either stored at −80 °C or processed as follows: centrifuge Oracol™ samplers at 491× *g*, 10 min at 10 °C and then at 1363× *g* for 15 min; supernatant separated from debris and transferred to 1.5 mL tubes.

Following the two previous centrifugations, the samples were centrifuged a final time at 1500× *g* for 3 min, supernatant transferred to fresh 1.5 mL microcentrifuge tubes, and stored at −80 °C. Samples were analyzed as described previously [6,20]. Briefly, the thawed saliva samples were diluted 1:4 in phosphate-buffered saline, 1% bovine serum albumin (PBS BSA) for a total of 50 µL and added to prewet 96-well filter plates (Millipore, MA, USA). Addition of an equal volume of 5 × 10^3^ beads from the combined bead sets brought the final dilution to 1:8. Antigen coupled beads were incubated with saliva dilutions for one hour at room temperature, in the dark, on a VWR™ microplate shaker (Radnor, PA, USA). Beads were washed with 100 l PBS BSA ×3 and incubated with a secondary anti-human biotin-labeled antibody for 1 h at room temperature with rotation as before. After incubation, the beads were washed again as described above and incubated with a streptavidin-phycoerythrin (SAPE) fluorescent reporter. After three final washes, the samples were analyzed on a Luminex 100 analyzer (Luminex™, Austin, TX, USA). Fluorescence intensity was expressed in median fluorescence intensity units (MFI).

### 2.2. Activation, Coupling, Controls and Measurements for Cross-Reactivity

Bead activation, antigen coupling, coupling confirmation, assay controls, and measurements of cross-reactivity have been described elsewhere [6,20]. In brief, beads were activated and coupled following manufacturer’s recommendations. An uncoupled bead set was used as a control to measure nonspecific binding and sample variability. Samples that showed levels of nonspecific binding to the uncoupled beads of ≥500 MFI were removed from further analyses because of possible contamination with serum or gum disease. Cross-reactivity was measured in monoplex, duplex and multiplex as described previously [13,20]. Characterized sera was used to assess the performance of the assay [20] and a signal-to-noise ratio was employed to determine the sensitivity of the assay as described [20,21].

### 2.3. Assessing Exposure

In line with approaches previously developed to examine biomarkers of exposure to environmental pathogens [15,20], our analyses focused chiefly on identifying the following parameters: immunoprevalence, co-immunoprevalence, immunoconversions and co-immunoconversions. Each of these parameters relates to the presence of circulating antibodies against specific pathogens and provide linked yet distinct information on exposure. Immunopositive samples are those with MFI values above the established cutoff defined as 10^mean(h)+3SD(h)^, where h = log_10_(MFI of uncoupled controls) [20]. Immunoprevalence (IP), is defined in the S1 samples collected on the beach and provides evidence of prior exposures. We defined an immunoconversion (IC) as the presence of detectable antibodies in biofluids, following infection [15]. IgG antibodies indicate an immune response to pathogens and serve as biomarkers of infection. We calculated and defined an immunoconversion as follows: S2 ≥ 4 × S1; S2 ≥ cutoff; S3 ≥ 3 × S1 [15]; S1–S3 are the sample periods. Traditionally, immunoconversions or seroconversions were defined as a four-fold increase from S1 to S2 [22,23,24,25] but we extended that definition by (a) ensuring that the S2 sample is immunopositive (MFI ≥ cutoff) and (b) adding a third sample (S3) to reduce the potential for false positives and accounting for IgG levels remaining elevated during the 40-day period after initial exposure. Co-immunoprevalence and co-immunoconversions (coinfections) are identified when a sample or an individual meets the IP or IC criteria, respectively, for more than one pathogen. Microsoft Excel 365, JMP 14 and MATLAB Release 2019b were used to perform data analyses.

### 2.4. Study Site

**Figure 1:**
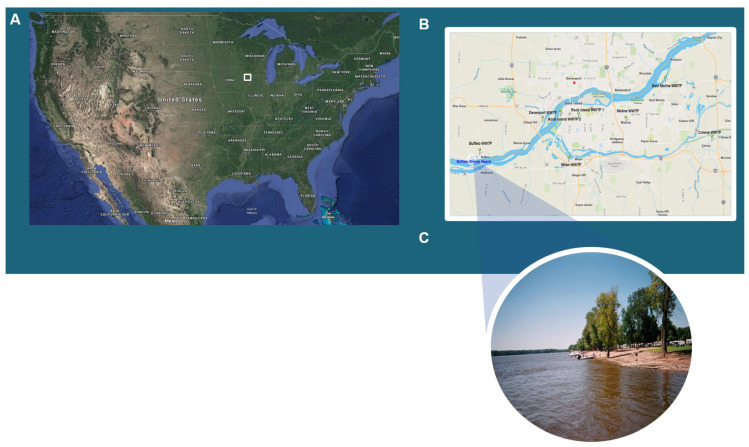
Maps of (**A**) the United States displaying the study area on the eastern border of Iowa. (**B**) The study area (lower left in dark blue) is along the Mississippi River downstream from Davenport, Iowa, and multiple wastewater treatment plants (WWTP). (**C**) A snapshot of the Buffalo Shores Beach recreational area.

Study subjects were recruited from Buffalo Shores Beach, a twenty-five-acre recreation area downstream from Buffalo, Iowa, and about 10 miles southwest of the city of Davenport on the Mississippi River (Figure 1A). Multiple wastewater treatment plants (WWTPs) located upstream from the beach (Figure 1B,C) may contribute to beach contamination. However, the main source of pollution appears to be the city of Davenport because, as shown in Table 2, that city has largest capacity and coincidentally did not chlorinate their secondary effluent before discharging it into the Mississippi River.

## 3. Results

**Figure 2:**
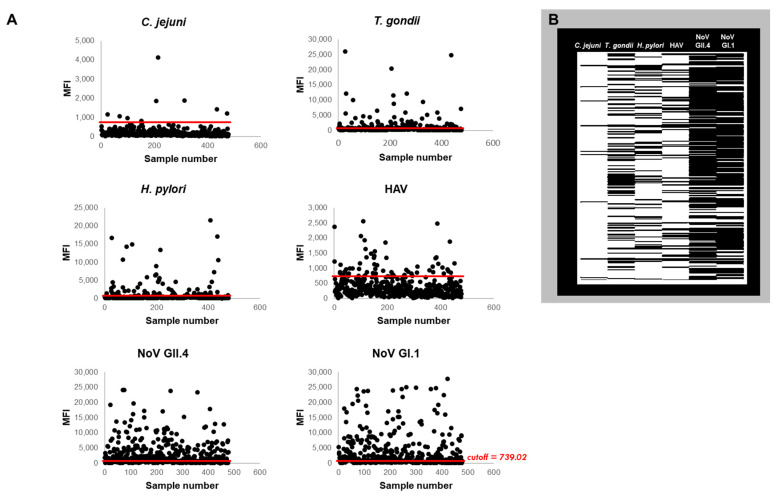
Immunoprevalence for targeted pathogens. (**A**) Median Fluorescence Intensity units (MFI) scatterplot showing all S1 samples with the cutoff (red line: 739.02 MFI) distinguishing positive (above) and negative (below) samples. (**B**) Immunoprevalence heatmap displaying the positive samples denoted by black lines.

We collected 840 saliva samples from 478 participants during the study period. The MFI values produced from applying the multiplex immunoassay to the samples and control beads are provided in Table S1. Five (5) samples were removed because they failed to meet the quality assurance/quality control criteria previously discussed (i.e., samples displayed nonspecific binding to the control beads at ≥500 MFI) [20]. The remaining samples (*n* = 835) were used in the analysis and include S1 (*n* = 478), and 357 (S2: *n* = 204; S3: *n* = 153, respectively). Figure 2 presents a summary of the MFI values and immunoprevalence status for each targeted pathogen with MFI values ranging from 7 (HAV) to 27819 (noroviruses). Figure 2A provides scatterplots of the baseline samples (S1) where positive samples are denoted as those with MFI values above the cutoff (red line: MFI ≥ 739.02). These positive samples are also visualized as black lines in the heatmap (Figure 2B). Table 3 compiles the immunoprevalence results by displaying details on individuals exposed to none, any, single or multiple pathogens (top and middle), as well as exposure rates to specific pathogens (bottom). Almost 80% of study participants had antibodies to at least one pathogen (mostly noroviruses: GII.4: 58.79%, GI.1: 59.41%) and over half (56.28%) showed evidence of prior exposure to multiple pathogens at the beginning of the study (Table 3).

**Figure 3:**
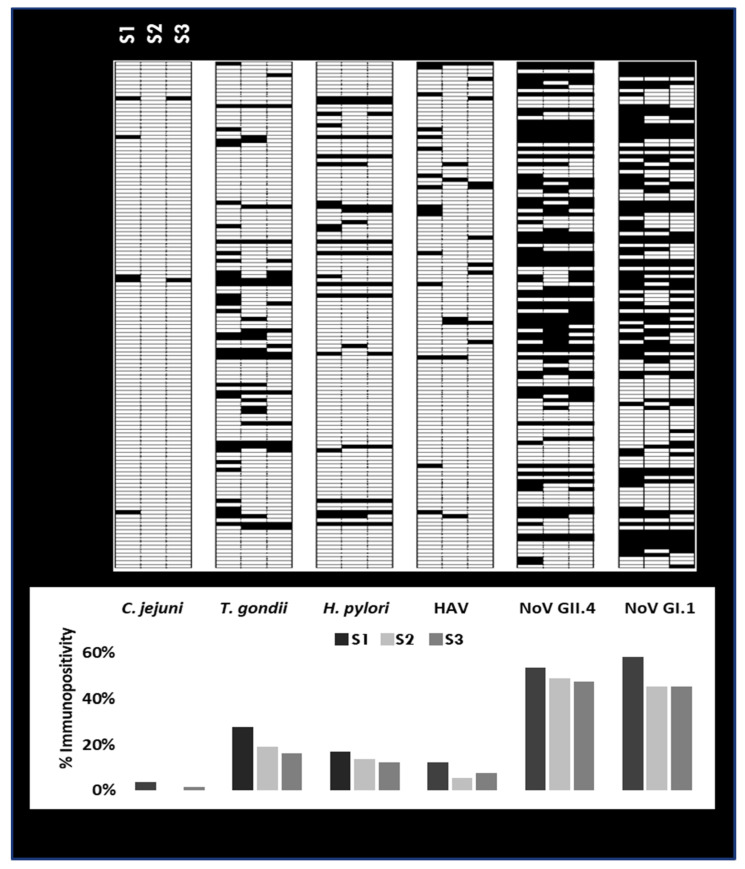
Immunopositivity summary: exposure status of the individuals who provided a saliva sample for each collection period (S1–S3). Upper panel: black lines represent the samples positive (MFI ≥ cutoff) for the targeted pathogens. Lower panel: % immunopositivity for the targeted pathogens.

During the initial collection period (S1), some individuals had antibodies to three or more (N ≥ 3) pathogens, however, most had antibodies to two (N = 2) or less with the most prevalent pair being amongst the noroviruses (45.19%) and between the noroviruses and *T. gondii* (with GI.1: 16.53%; with GII.4: 13.60%) (Table 3 and Table 4). Only 131 individuals (*n* = 131) provided samples for all three periods; hence, this cohort was used to examine MFI patterns over time to detect immunoconversions (IC). The immunopositivity summary displays the exposure status (upper panel: black line = positive) and percent immunopositivity (lower panel) for each sample period (Figure 3). Results showed that the beachgoers were primarily exposed to noroviruses and *T. gondii* and exposure rates tended to decline over time.

**Figure 4:**
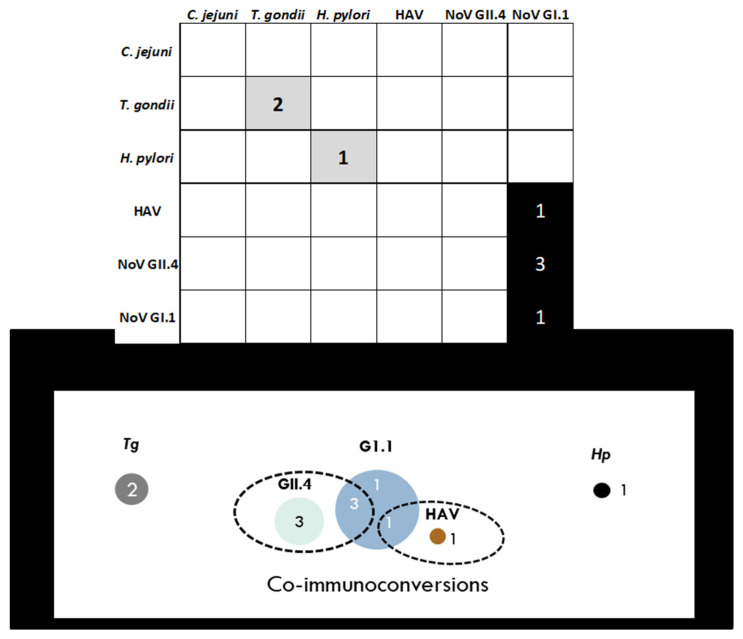
Immunoconversion summary denoting the number of immunoconversions and co-immunoconversions to the pathogens under study. Note *Tg*: *T. gondii*; GI.1: norovirus GI.1; GII.4: norovirus GII.4; HAV: hepatitis A virus and *Hp*: *H. pylori*.

Based on the IC criteria (S2 ≥ 4 × S1; S2 ≥ cutoff; S3 ≥ 3 × S1), 8 individuals (6.11%) immunoconverted to at least one pathogen (NoV GI.1: 5 (3.82%); NoV GII.4: 3 (2.29%), *T. gondii*: 2 (1.53%); hepatitis A virus (HAV): 1 (0.76%); *H. pylori*: 1 (0.76%)) and half of those (*n* = 4) immunoconverted to two pathogens (Table 5). Figure 4 summarizes the immunoconversions including a visualization of the three co-immunoconversions between the noroviruses and one between NoV GI.1 and HAV. No immunoconversions were observed for *C. jejuni*.

**Figure 5:**
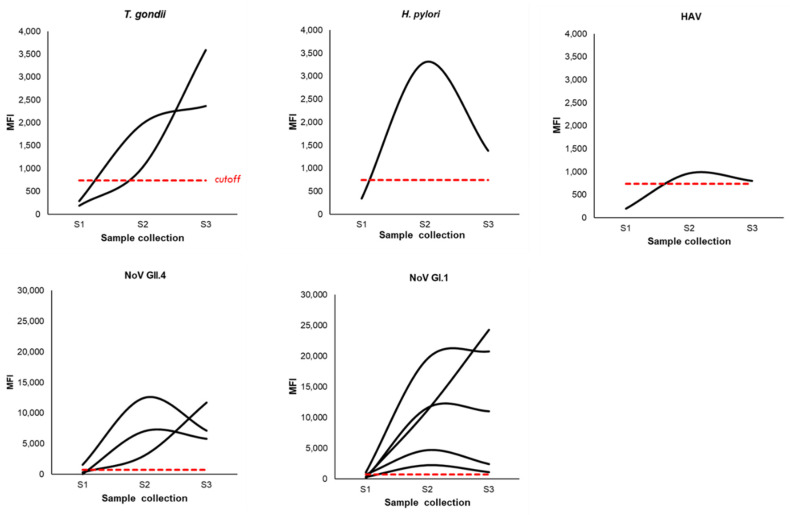
MFI curves for the pathogens under study. Plots showing IgG in MFI from S1 to S3 for immunoconversions. Red dashed line = cutoff (739.02 MFI).

MFI trends over time reflect the IgG antibody responses during the immunoconversions (Figure 5). Most of the immunoconversions begin with a negative baseline sample, after which the MFI value increased to above the cutoff in S2 (positive) and then declined (remaining positive) at S3.

## 4. Discussion

In this study, we applied a bead-based salivary antibody immunoassay in a multiplex format to determine antibody prevalence to select waterborne pathogens among a group of beachgoers in Buffalo Shores Beach, IA. There was possible contamination at the beach due to the number of WWTP discharging into the area with the largest one in Davenport, IA not providing any chlorination (Figure 1, Table 2). Immunoassay results indicated that: nearly 80% of the beachgoers had antibodies to at least one of the targeted pathogens; about three-fifths had baseline exposures (immunoprevalence) to noroviruses; at least 12% were exposed to *T. gondii*, *H. pylori* and hepatitis A virus; and antibodies against *C. jejuni* were rare. Generally speaking, these prevalence rates are in line with data previously reported in the literature [13,22,25,26,27,28,29]. As expected, previous exposure to noroviruses was the most common (co-immunoprevalence = 45.19%; co-immunoconversions or coinfections: 2.29%), however multiple immunoconversions (incident infections) were also found amongst other pathogens with the second and third largest pairing being between *T. gondii* and both NoV GI.1 (16.53%) and NoV GII.4 (13.60%).

Many of these findings are similar to results from our previous study at Boquerón Beach, Puerto Rico (PR) [13]. Although overall immunoprevalence was generally higher in Iowa, prior exposures in Boquerón Beach were also largely to noroviruses (GI.1: 48.6%; GII.4: 37.6%) and hepatitis A (Iowa: 12.55%, PR: 16.17%). We found similar levels of immunoprevalence to *H. pylori* (Iowa 14.85%, PR: 14%) and *C. jejuni* (Iowa: 1.88%, PR: 2.26%). As expected, given its high prevalence, incident infections were primarily to noroviruses for both communities. There was a higher immunoprevalence for *T. gondii* in Iowa (Iowa: 22.8%, PR: 2.26%), however the immunoprevalence we observed in Boquerón Beach, Puerto Rico is considerably lower than has been observed by others. For example, a recent cross-sectional study by Gonzalez-Pons et al. found a seroprevalence rate of 33% for *H. pylori* in Puerto Rico, more than twice the rate we found [30]. This disparity may be attributed to the differences in our study designs, approaches and study population characteristics. While our Boquerón Beach studies focused specifically on using a multiplex immunoassay to detect salivary antibodies to six targeted pathogens in our beachgoer population, Gonzalez-Pons et al. used a representative sample of archived, frozen serum samples from an existing population-based biorepository to estimate *H. pylori* exposure using logistic regression [13,15,18,30]. Additionally, our samples were collected from individuals recreating at Boquerón Beach during the summer of 2009 and Gonzales-Pons et al. gathered data from a 2005–2008 survey study that recruited noninstitutionalized individuals in a stratified, multistage, probability cluster design of all households in Puerto Rico [30].

There are several limitations in applying immunoassays in population studies. First, detecting antibodies and other analytes in biological samples is critical for many applications but most immunoassays suffer from background noise that limit the sensitivity and dynamic range of the assay [31]. Secondly, participant age plays an important role in antibody responses. According to Weiskopf et al., 2009, as people get older, the immune system declines causing immunosenescence that leads to increased susceptibility to severe infectious diseases and low efficacy of vaccination [32]. Antibody responses in children, on the other hand, may be short-lived. These responses may also be at a lower level or altogether absent as is the case with *Plasmodium falciparum* infection which causes malaria [33]. These anomalies may lead to either overestimation or underestimation of antibody responses in these populations. Thirdly, while it is practically impossible to eliminate all antibody cross-reactivity, every effort has been made to reduce and account for background signal. Fourthly, as shown in a recent paper, HAV immunoconversions may be underestimated in the present study because it was measured at S2 (10 days after beach activity) [18]. That period may be too short to properly assess antibody responses since the virus has a 14–49-day incubation period [34]. Preliminary (unpublished) analyses of water samples and epidemiological data showed evidence of fecal contamination, but no significant association between gastrointestinal symptoms and water-related activity at the beach [2]. This result is similar to the Boquerón Beach study where the infections detected through application of the immunoassay were found to be asymptomatic [16]. Finally, researchers have found seasonal and circadian patterns in antibody titers [35] and such patterns may have affected the antibody levels we observed, particularly in the assessment of immunoconversions. Our study design did not account for these circadian patterns because the S2 and S3 samples were self-collected. Furthermore, it was outside of the scope of our study to control for time of sample collection. However, our approach is consistent with other population-based salivary antibody surveys [36,37]. Further, salivary IgG has long been used to screen for HIV, hepatitis A virus, dengue, and many other pathogens without control for time of collection [38,39,40].

Despite the limitations, the multiplex immunoassay is based on examining antibodies as biomarkers of exposure and is useful for public health studies regardless of the route or source of exposure (e.g., food, water, environment). In comparison to approaches that measure analytes one at a time, the multiplex immunoassay approach afforded the opportunity to analyze multiple parameters simultaneously. Hence, we were able to observe coinfections and prior infections to multiple pathogens at the same time; valuable observations that would not have been possible by monitoring one pathogen at a time (Figure 2, Figure 3, Figure 4 and Figure 5; Table 3, Table 4 and Table 5). The development of a common cut-off for the entire multiplex, also contributed to illuminating and simplifying the prevalence of antibodies circulating in the community. The heatmaps in Figure 2 and Figure 3 provide an additional level of data visualization regarding the positivity for each pathogen and collection time points, respectively. This immunoassay serves as a valuable screening tool to measure immunoprevalence, co-immunoprevalence, incident infections and coinfections occurring in the population.

## 5. Conclusions

Assessment of the saliva samples collected from the Iowa riverine beachgoers provided evidence of exposure to the six waterborne pathogens analyzed. The initial (S1) samples presented important information about circulating antibodies in the population from previous infections of unknown origin to each of the studied pathogens. In addition to identifying these pathogens as etiological agents of prior infections in the population, we observed new or incident infections in some of the study subjects possibly resulting from their activity at the study site. These health effects data are critical to the development of mitigation efforts to protect human health and the environment. Antibody studies, such as the one we present here, provide valuable information on the prevalence of exposure and incident infections which is useful for public health officials, policy makers, risk assessors and epidemiologists concerned with understanding the etiology of infections for evaluating and mitigating the health effects of microbiological exposures. Such information could assist in the early detection of outbreaks such as the current COVID-19 pandemic and help determine the penetration of these pathogens in the population regardless of symptomology. Future studies involve applying the assay to known and emerging pathogens (e.g., *Legionella pneumophila*, SARS-CoV-2) and the comparison of community exposure patterns.

## Figures and Tables

**Table 1:** Multiplex immunoassay reagents, commercial sources, and concentrations.

Organism	Antigen (Ag)	Source	Amt. of Ag Coupled (µg)
Hepatitis A virus	Cell culture concentrate	Meridian	100
Norovirus GI.1	P-particle	Xi Jiang *	5
Norovirus GII.4	P-particle	Xi Jiang *	5
*Campylobacter jejuni*	Heat-killed whole bacterial cells	KPL	50
*Helicobacter pylori*	Bacterial cell lysate	Meridian	25
*Toxoplasma gondii*	Recombinant p30 (SAG1)	Meridian	25

* Jiang Lab, Cincinnati Children’s Hospital, Cincinnati, OH, USA. Ag refers to antigen type. Source references where antigens were purchased. Amt. of Ag Coupled denotes the concentration of antigens coupled to each bead set. KPL refers to Kirkegaard Perry Labs; SAG-1 (Surface Antigen-1).

**Table 2:** Communities contributing sewage (in gallons per day: GPD) to the Mississippi River upstream of Buffalo Shores Beach.

Community	Population	Plant Capacity	Treatment	Chlorination
Moline, IL, N ^1^	43,483 *	1,375,000 GPD	Secondary	Yes
Rock Island, IL	39,018	16,000,000 GPD	Tertiary	Yes
Milan, IL	5099	1,000,000 GPD	Secondary	Yes
Moline, IL, S ^1^	43,483 *	1,800,000 GPD	Secondary	Yes
Colona, IL	5099	1,000,000 GPD	Secondary	Yes
East Moline, IL	21,302	11,100,000 GPD	Secondary	Yes
Davenport, IA	127,142	20,000,000 GPD	Secondary	No
Buffalo, IA	1260	130,000 GPD	Secondary	Yes

Source: Information obtained from the various community websites at the time study began. * only includes half of the Moline, IL population. ^1^ N indicates the north sewage treatment plant. S indicates the south sewage treatment plant.

**Table 3:** Immunoprevalence summary provides an overview of the baseline (S1) exposure to the pathogens under study. Top: individuals with exposure to none, any, single or multiple pathogens. Middle: individuals with exposure to N (0 to 6) pathogens, where N ≥ 2 denotes the individuals with exposures to multiple pathogens. Bottom: individuals with exposure to specific pathogens.

Immunoprevalence	*n* (%)	*n*	%
None	102 (21.34)	102	21.34%
Any (N ≥ 1)	376 (78.66)	376	78.66%
Single (N = 1)	107 (22.38)	107	22.38%
Multiple (N ≥ 2)	269 (56.28)	269	56.28%
To N pathogens			
N	*n* (%)	*n*	%
0	102 (21.34)	102	21.34%
1	107 (22.38)	107	22.38%
2	149 (31.17)	149	31.17%
3	82 (17.15)	82	17.15%
4	29 (6.07)	29	6.07%
5	7 (1.46)	7	1.46%
6	2 (0.42)	2	0.42%
To specific pathogens			
Pathogen	*n* (%)	*n*	%
*C. jejuni*	9 (1.88)	9	1.88%
*T. gondii*	109 (22.80)	109	22.80%
*H. pylori*	71 (14.85)	71	14.85%
Hep. A	60 (12.55)	60	12.55%
NoV GII.4	281 (58.79)	281	58.79%
NoV GI.1	284 (59.41)	284	59.41%

Note: “*n*” denotes number of individuals and “N” denotes number of pathogens.

**Table 4:** Co-immunoprevalence: Number and percentage of individuals with samples that are positive to two (N = 2) pathogens at S1.

	*C. jejuni*	*T. gondii*	*H. pylori*	HAV	NoV GII.4	NoV GI.1
*C. jejuni*		4 (0.84%)	5 (1.05%)	5 (1.05%)	8 (1.67%)	8 (1.67%)
*T. gondii*			29 (6.07%)	23 (4.81%)	65 (13.60%)	79 (16.53%)
*H. pylori*				11 (2.30%)	50 (10.46%)	61 (12.76%)
HAV					52 (10.88%)	53 (11.09%)
NoV GII.4						216 (45.19%)
NoV GI.1						

**Table 5:** Immunoconversions (IC): number (*n*) and percentage (%) of individuals who immunoconverted to the pathogens based on the established criteria: S2 ≥ 4 × S1; S2 ≥ cutoff; S3 ≥ 3 × S1.

Immunoconversions	*n* (%)	*n*	%
None	123 (93.89)	123	93.89%
Any (N ≥ 1)	8 (6.11)	8	6.11%
Single (N= 1)	4 (50)	4	50%
Multiple (N ≥ 2)	4 (50)	4	50%
To N pathogens			
N	*n* (%)	*n*	%
0	123 (93.89)	123	93.9%
1	4 (3.05)	4	3.05%
2	4 (3.05)	4	3.05%
3	0 (0.00)	0	0%
4	0 (0.00)	0	0%
5	0 (0.00)	0	0%
6	0 (0.00)	0	0%
To specific pathogens			
Pathogen	*n* (%)	*n*	%
*C. jejuni*	0 (0.00)	0	0%
*T. gondii*	2 (1.53)	2	1.53%
*H. pylori*	1 (0.76)	1	0.76%
HAV	1 (0.76)	1	0.76%
NoV GII.4	3 (2.29)	3	2.29%
NoV GI.1	5 (3.82)	3	3.82%

## Data Availability

Data is contained within the Supplementary Material. The data presented in this study are available in www.mdpi.com/xxx/s1: Iowa data set.

## Supplementary Materials

The following are available online at https://www.mdpi.com/article/10.3390/ijerph18115797/s1. Table S1: Iowa data set.
